# Associations between Season and Gametocyte Dynamics in Chronic *Plasmodium falciparum* Infections

**DOI:** 10.1371/journal.pone.0166699

**Published:** 2016-11-21

**Authors:** Amal A. H. Gadalla, Petra Schneider, Thomas S. Churcher, Elkhansaa Nassir, Abdel-Muhsin A. Abdel-Muhsin, Lisa C. Ranford-Cartwright, Sarah E. Reece, Hamza A. Babiker

**Affiliations:** 1 Department of Biochemistry, Faculty of Medicine and Health Sciences, Sultan Qaboos University, Muscat, Oman; 2 Department of Molecular Epidemiology, Tropical Medicine Research Institute, National Centre for Research, Khartoum, Sudan; 3 Institutes of Evolution, Immunology and Infection Research, University of Edinburgh, Edinburgh, United Kingdom; 4 Department of Infectious Disease Epidemiology, Imperial College London, London, United Kingdom; 5 Medicinal and Aromatic Plants Research Institute and Traditional Medicine, National Centre for Research, Khartoum, Sudan; 6 Department of Biology, Faculty of Science, University of Hail, Baqaa, Kingdom of Saudi Arabia; 7 Institute of Infection, Immunity and Inflammation, College of Medical, Veterinary and Life Sciences, University of Glasgow, Glasgow, United Kingdom; 8 Centre for Immunity, Infection & Evolution, School of Biological Sciences, University of Edinburgh, Edinburgh, United Kingdom; Institut Pasteur, FRANCE

## Abstract

**Introduction:**

In a markedly seasonal malaria setting, the transition from the transmission-free dry season to the transmission season depends on the resurgence of the mosquito population following the start of annual rains. The sudden onset of malaria outbreaks at the start of the transmission season suggests that parasites persist during the dry season and respond to either the reappearance of vectors, or correlated events, by increasing the production of transmission stages. Here, we investigate whether *Plasmodium falciparum* gametocyte density and the correlation between gametocyte density and parasite density show seasonal variation in chronic (largely asymptomatic) carriers in eastern Sudan.

**Materials and Methods:**

We recruited and treated 123 malaria patients in the transmission season 2001. We then followed them monthly during four distinct consecutive epidemiological seasons: transmission season 1, transmission-free season, pre-clinical period, and transmission season 2. In samples collected from 25 participants who fulfilled the selection criteria of the current analysis, we used quantitative PCR (qPCR) and RT-qPCR to quantify parasite and gametocyte densities, respectively.

**Results and Discussion:**

We observed a significant increase in gametocyte density and a significantly steeper positive correlation between gametocyte density and total parasite density during the pre-clinical period compared to the preceding transmission-free season. However, there was no corresponding increase in the density or prevalence of total parasites or gametocyte prevalence. The increase in gametocyte production during the pre-clinical period supports the hypothesis that *P*. *falciparum* may respond to environmental cues, such as mosquito biting, to modulate its transmission strategy. Thus, seasonal changes may be important to ignite transmission in unstable-malaria settings.

## Introduction

In many areas, malaria transmission is seasonal and limited to a short window of time, often a few months, depending on availability of mosquito vector breeding habitats [1–3], temperature and relative humidity [4–6]. During the transmission-free dry season, *Anopheles* mosquitoes are often undetectable or at extremely low density and confined to a few refugia. This occurs in endemic areas across the Sahel [7], Sudan [2,8], Senegal [1], Burkina Faso [9] and The Gambia [10]. Following the start of annual rains, vector densities increase rapidly [10], suggesting that they persist locally, possibly via aestivation [8], or appear after long-distance migration [11,12]. However, it takes at least 2 months after the start of rains for the appearance of new malaria cases [2,13]. The gap between the revival of mosquitoes and appearance of clinical cases of *P*. *falciparum*—here denoted the pre-clinical period—is observed in areas with a short transmission season, such as eastern Sudan [1,2,10].

Malaria transmission depends on the ability of the parasite to produce the sexual stages (gametocytes) that infect mosquitoes, which in turn develop to produce the stages transmissible to the next vertebrate host (sporozoites). Throughout infections, a variable proportion of asexual stages commit to gametocytogenesis [14,15]. In *P*. *falciparum*, immature gametocytes remain sequestered in deep tissues for approximately 10 days before appearing as morphologically distinct gametocytes in the peripheral blood [16,17] with a lifespan of approximately 6.5 days on average (range 1.3–22.2 days) [18–20]. Gametocyte investment in *Plasmodium* species is thought to be adjusted by parasites in response to the prevailing within-host environment, to balance between-host transmission and within-host survival [21–23]. Several factors modulate gametocyte investment in *P*. *falciparum* including parasite density [14,24,25], multiplicity of infection [26], anaemia [25,27,28], duration of infection [29], host immunity [30], and anti-malarial drugs [31–33].

Whether *P*. *falciparum* modulates gametocyte investment in response to the appearance of vectors at the start of a new transmission season is unknown. Studies on rodent models have yielded contradictory results for whether malaria parasites respond to probing by mosquitoes by increasing gametocyte investment [34,35]. A more recent experiment using an avian malaria parasite species revealed that parasites responded to mosquito biting by enhancing transmission, but whether this involved an increase in gametocyte investment is unknown [36]. If *P*. *falciparum* parasites adjust their transmission strategy in response to the availability of vectors, this could significantly impact malaria transmission dynamics, particularly in seasonal malaria settings, by enabling parasites to maximise transmission opportunities.

Here, we study naturally occurring human infections of *P*. *falciparum* in an area of distinct seasonal transmission in eastern Sudan. Our aim is to assess whether gametocyte production shows seasonal variation coinciding with the limited duration for transmission. Specifically, we hypothesise that if parasites respond to, or prepare for, the start of the transmission season, then gametocyte density and the slope of the correlation between gametocyte density and parasite density (i.e. the proportion of parasites that are gametocytes) will increase during the pre-clinical period compared to the preceding transmission-free season.

## Methods

### Study area

The study was carried out in Asar village (longitude 358.300 E and latitude 138.300 N), Gedaref state, eastern Sudan. *P*. *falciparum* is the predominant malaria parasite, accounting for more than 90% of all malaria infections. *Anopheles arabiensis* is the main mosquito vector. The entomological inoculation rate is less than one bite per person during the transmission season [37].

### Characterisation of seasons

**Fig 1:**
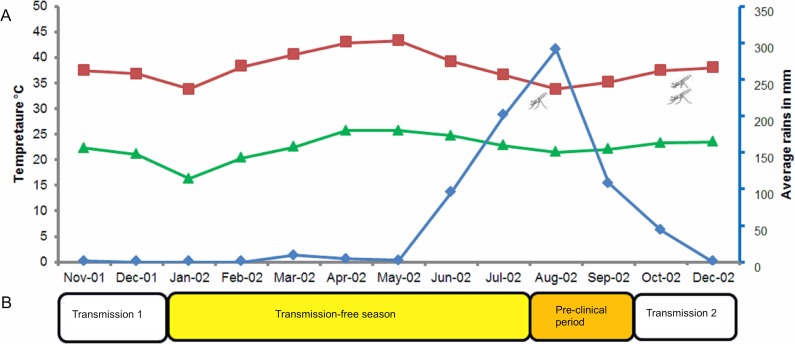
Seasonality of annual rains, mosquito abundance, and categorisation of seasons. (A) Monthly average rainfall in mm (blue line), maximum (red line) and minimum (green line) temperature in the study area between November 2001 and December 2002 (Meteorological Authority, Sudan). Mosquito symbols indicate the expected appearance of mosquitoes (July 2002) and peak mosquito densities (October 2002). (B) Distinct epidemiological phases of malaria transmission; transmission season 1 (November to December 2001), transmission-free season (January to July 2002), pre-clinical period (August and September 2002) and transmission season 2 (October and December 2002).

We defined four distinct seasons based on a combination of records for temperature, rainfall, humidity, *Anopheline* abundance and incidence of malaria cases as described by previous studies at the study site [2,37]. *Anopheles* mosquitoes generally appear in late July to early August reaching peak densities in late September/October. Malaria cases are usually reported between late September and November with a peak in October [2,37]. By January, the number of malaria cases drops substantially. Previous entomological surveys have shown no evidence of transmission during the long dry season [2,37]. The pattern of rainfall during the study period was as the following: in November 2001 (the start of study recruitment), the monthly average rainfall was 1.2 mm/month. There was no rain detected between December 2001 and February 2002. In March, April and May 2002 the average rainfall was 9.4, 4.6 and 2.2 mm/month, respectively. The main annual rains started in June 2002, with an average rainfall of 95.7 mm/month, and increased to reach a peak of 290 mm/month in August 2002 (Fig 1A). The highest and lowest temperatures recorded during the study period were 43.3°C (May 2002) and 21.0°C (August 2002), with the latter coinciding with the peak of the rains (Fig 1A). The temperature increased again in September to a maximum of 37°C, which combined with the rains to increase humidity, creating optimum conditions for mosquito breeding and larval development [5]. Therefore, for our study period we defined four seasons of epidemiological importance as: (a) transmission season 1 (November and December 2001) with mosquitoes present and malaria cases reported; (b) transmission-free season (January to late July 2002) without *Anopheles* mosquitoes or malaria cases [2]; (c) pre-clinical period (August to late September 2002) with the presence of *Anopheles* mosquitoes but before clinical cases are reported [2]; and (d) transmission season 2 (October to December 2002), with the presence of *Anopheles* mosquitoes and clinical cases [2] (Fig 1B).

### Study design, subjects and samples

The details of enrolment of subjects, sample collection and processing are reported in [26]. Briefly, during November 2001 (start of transmission season 1), a cohort of 123 symptomatic malaria patients, aged ≥ 10 years, with positive *P*. *falciparum* blood films was recruited to the study and treated with chloroquine and/or sulfadoxine/pyrimethamine according to malaria treatment guidelines at the time of the study [26]. Venous blood (2 ml) was collected in heparinised tubes from each participant before treatment and during the monthly follow up visits until December 2002 (except November 2002) [26]. Blood samples were centrifuged, plasma was separated from the blood cells at the study site and samples were immediately preserved in liquid nitrogen and transported to University of Edinburgh, where they were kept at -80°C. Ethical clearance for sample collection in 2001 was obtained from the Ethical Committee of the Ministry of Health, Sudan. Blood samples were collected with written or oral informed consent of all patients or their parent/ gradient. For the current analysis no ethical approval was sought, as samples were archive samples [26].

### Exclusion/inclusion criteria

Of the 123 recruited participants, 25 individuals with the following infection characteristics were included in the present study:

(1) Sustained chronic sub-microscopic parasite densities, determined as at least 3 time points during the study that were parasite-positive by qPCR. Fourteen (56%) participants had 7 to 12 positive time points, 5 (20%) participants had 5 to 6 positive time points and 6 (24%) participants had 3 to 4 positive time points. (2) No evidence of new infections during the pre-clinical period (August and September); in terms of either sudden appearance of high parasite density or appearance of new parasite clone. (3) No self-treatment with anti-malarials during the period between June and September, to rule out any possible effect of drug on gametocyte density during the pre-clinical period. (4) Participants who received treatment (n = 2) or who developed new infections (identified by presence of new alleles and high parasitaemia) during transmission season 2 were not excluded from the analysis because their gametocyte density during the transmission-free season and the pre-clinical period could not be affected by future drug treatment.

Over the follow up period we analysed 270 samples from the 25 eligible participants. There were 30 missing samples across 12 different time points. For the current analysis, only data from samples collected after drug treatment were described (i.e. samples collected at enrolment in November 2001 were excluded).

### Quantification of parasite and gametocyte densities

The total number of parasites in the samples was quantified from DNA extracted in 2002 (using the Chelex method) from blood spotted onto filter paper [26]. Only 64 time points had missing DNA samples; for those samples DNA was re-extracted in 2012 from stored frozen blood cells using the QIAamp DNA mini kit (Qiagen). *P*. *falciparum* 18S ribosomal RNA gene copy number was quantified by qPCR as described in [38], except using TaqMan Universal PCR Master Mix (ThermoFisher) and probe concentrations reduced to 100nM. 18S copy numbers were converted to parasite numbers using a calibration curve generated from *P*. *falciparum* clone 3D7 parasite DNA, a range of 0.014–138938 parasites/μl of DNA. The 18S qPCR amplification efficiency was 97.7% (s.e. 0.01%), the inter-assay variability (as a measure of qPCR reproducibility, but not uncertainty that may arise from blood sampling low level of parasitaemia [39]) between standard curves efficiency was <5% at all densities and the correlation between log_10_ parasite numbers and Cq values was significant (adjusted R^2^ >0.98 for all PCRs with P <0.001). The lower limit of detection was 0.139 parasites/μl of DNA corresponding to 0.1 parasites/μl of blood. Total parasite density was quantified in duplicate for each sample and the correlation between duplicate Cq values was significant (R^2^ = 0.95, P <0.001).

To quantify gametocytes, RNA was extracted in 2002 using the High Pure RNA isolation kit (Roche) [26,40] and stored at -80°C until used for the current study. RNA was extracted for a few missing RNA samples using the SV Total RNA Isolation System (Promega, UK) in 2012. Extracted RNA was converted to cDNA using the High Capacity cDNA Reverse Transcription Kit (ThermoFisher, UK). From the cDNA, expression of the *pfs25* (female gametocyte specific) and *pfs230p* (male gametocyte specific) genes were quantified by PCR as described in [41], with the exception that a TaqMan probe (6FAM-ACTGGAATCGAACAACA-MGB, 250nM) and FastStart Taq dNTPack (Roche) were used to quantify male gametocytes. Levels of *pfs25* and *pfs230p* in the cDNA samples were converted to female and male gametocyte counts, respectively, using calibration curves of *in vitro* DNAs (PCR amplified sequence of DNA spanning the region of the qPCR amplicons) and their relationship to *P*. *falciparum* female and male gametocyte counts from *in vitro* culture [41]. The female and male gametocyte RT-qPCR assays performed with amplification efficiencies of 94.2% (s.e. 0.03) and 89.6% (s.e. 0.02) respectively. Inter-assay variability for both assays was <6% at all densities, and log_10_ male or female gametocyte numbers and Cq values were significantly correlated (adjusted R^2^ >0.98 for all PCRs with P <0.001). The correlation between duplicate quantification of the same samples was strong (R^2^ = 0.950, P <0.001; 10% of samples randomly tested). Total gametocyte numbers were calculated by summing the male and female gametocytes, with a lower limit of detection of 0.5 gametocytes/μl of blood.

Because DNA was extracted from unknown amounts of blood spotted on filter paper and RNA was extracted from 50uL of blood cells (after plasma removal), the association to full blood volumes could not be simply determined. Therefore, absolute quantification of parasite- and gametocyte densities per μl of blood was not possible. Instead, parasite and gametocyte counts were normalised against human glyceraldehyde-3-phosphate dehydrogenase (GAPDH). GAPDH was quantified as described in [42], with a modified reverse primer (SCTGGCGACGCAAAAGA; S = G or C). GAPDH qPCR and RT-qPCR reaction volumes, mastermixes and probe concentrations were the same as those for the 18S PCR and *pfs25* RT-PCR, respectively. Between-individual potential differences in GAPHD, were controlled for by using patient identification (ID) (categorical variable) as a random effect in the statistical analysis.

### Statistical analysis

Generalized linear mixed models (GLMMs) [43] were used to estimate the changes in parasite prevalence, total parasite density, gametocyte prevalence and gametocyte density across seasons. These parameters were used as response variables in different models and allowed to vary between participants by including participant ID as a random effect. This, both increases the ability to detect changes over time as there is substantial inter-participant variability, and also accounts for the missing time points by supplying a predicted value. Total parasite density and gametocyte density were analysed on the same scale in which they were estimated (i.e. logarithm to base 10) to reduce biases caused by diagnostic measurement error. Sample processing differences and different WBC reference samples (healthy volunteers from Oman (n = 5) for DNA and from the UK (n = 10) for RNA) that were used for normalisation of gametocyte and parasite counts resulted in relative quantification differences between gametocytes (RNA) and total parasites (DNA) from the same blood sample. Whereas parasite and gametocyte dynamics for each participant can be followed over time, absolute densities of parasites and gametocytes of the same sample are not directly comparable. The relative difference would lead to the appearance that some samples had a greater number of gametocytes than total parasites. Therefore both gametocyte and total parasite densities are described as arbitrary units, denoted as parasites or gametocytes /μL blood_DNA_ or /μL blood_RNA_ to emphasize these are not absolute quantities and are not directly comparable between DNA and RNA samples.

A variety of error distributions (negative binomial, zero-inflated negative binomial, zero-inflated Poisson and zero-inflated normal) were tested to describe patterns for total parasite density and gametocyte density. A zero-inflated normal distribution gave the most parsimonious fit and was used for both of the continuous dependent variables. Parasite prevalence and gametocyte prevalence were fitted to binomial distribution models. Models were built using forward stepwise selection, and Log likelihood Ratio Tests (LRT) were used to determine the most parsimonious model. If more than one model fitted the data, the one with the lowest Akaike Information Criterion (AIC) was selected. R program version 3.0.1 and glmmADMB package [44] were used for the analysis.

For parasite prevalence and density models, fixed effect variables included season (categorical variable), and for gametocyte prevalence and density models fixed effect variables included season (categorical variable), total parasite density, and their interactions. The most parsimonious models included season (for parasite prevalence and parasite density); total parasite density (for gametocyte prevalence); and season, total parasite density and the interaction between them (for gametocyte density).

## Results

### Seasonal variation in parasite prevalence and density

Parasite prevalence and density data are presented in Table 1, alongside the estimates from our statistical models, in which between- participant variation has been controlled for. The average estimated parasite prevalence among the participants (n = 25) was 81.3% (s.e. = 2.48) one month after drug treatment at the end of transmission season 1 (December 2001). Prevalence varied across the seasons ($X_{3}^{2}$ = 8.966, P = 0.029). The average estimated prevalence decreased significantly in the transmission-free season ($X_{1}^{2}$ = 7.71, P = 0.005) compared to December 2001, and stayed at a similar level during the pre-clinical period ($X_{1}^{2}$ = 0.64, P = 0.423) compared to the transmission-free season ($X_{1}^{2}$ = 0.318, P = 0. 0.573; Table 1). There was no significant difference in parasite prevalence between the pre-clinical period and transmission season 2 ($X_{1}^{2}$ = 0.186, P = 0.666; Table 1).

**Table 1:** Prevalence and densities of total parasites and gametocytes in samples taken from 25 individuals selected during transmission 1.

Season	Transmission 1	Transmission-free	Pre-clinical	Transmission 2
	(Only data from December 2001)	January to July 2002	August and September 2002	October and December 2002
**Presence of *Anopheles* mosquitoes [2,7]**	Yes	No	Yes	Yes
**Presence of clinical cases [2]**	Yes	No	No	Yes
**Number of samples**^**a**^	25	152	47	46
**Data summary**^**b**^				
**Parasite prevalence**	20 (80.0%)	83 (54.6%)	23 (48.9%)	25 (54.3%)
**Number of individuals with at least one positive sample**^**c**^	20 (80.0%)	25 (100%)	20 (80.0%)	17 (68.0%)
**Parasite density /μL blood**_**DNA**_^**d**^	1.84 (0.26)	0.70 (0.08)	0.52 (0.11)	0.92 (0.18)
**Gametocyte prevalence**	13 (52.0%)	50 (32.9%)	10 (21.3%)	12 (26.1%)
**Number of individuals with at least one gametocyte positive sample**^**c**^	13 (52%)	20 (80.0%)	8 (32.0%)	11 (44.0%)
**Gametocyte density /μLblood**_**RNA**_^**d**^	1.10 (0.25)	0.47 (0.07)	0.44 (0.15)	0.29 (0.10)
**Model estimated values**^**e**^				
**Parasite prevalence**^**d**^	81.3% (2.48)	54.67% (1.64)^**f**^	48.7% (2.97)	54.4% (2.98)
**Parasite density (/μL blood**_**DNA**_**)** ^**d**^	1.63 (0.04)	0.68 (0.01)^**f**^	0.52 (0.03)	0.97 (0.03)^**f**^
**Gametocyte prevalence**^**d**^	45.9% (4.35)	29.8% (1.67)	25.4% (2.03)	33.1% (3.081)
**Gametocyte density (/μL blood**_**RNA**_**)**^**d**^	0.89 (0.04)	0.38 (0.01)^**f**^	0.62 (0.05)^**f**^	0.28(0.03)^**f**^

^a^Total number of samples collected during each season from the 25 participants

^b^ Raw data are presented to describe the infection parameters as observed among the study cohort.

^c^ Number of participants with at least one positive sample during a season.

^d^ Mean (continuous variables) or percentage (categorical variables) and standard error (s.e.) are presented.

^e^Model estimates are shown, which are more informative than raw data because of inter-participant variation and, for gametocyte estimates, also the effect of seasonal variation in parasite densities have been controlled for.

^f^Significant difference compared to the previous season

The average estimated parasite density was 1.63 log_10_ (s.e. = 0.04) parasites/μl blood_**DNA**_ after drug treatment during transmission season 1, i.e. December 2001. Parasite density varied across the seasons ($X_{3}^{2}$ = 74.08, P < 0.001). Specifically, average estimated parasite density decreased during the transmission-free season ($X_{1}^{2}$ = 24.33, P < 0.001) compared to December 2001 (Table 1). The average estimated parasite density remained similar between the transmission-free season and the pre-clinical period ($X_{1}^{2}$ = 0.99, P = 0.320) and increased from the pre-clinical period to the transmission season 2 ($X_{1}^{2}$ = 5.08, P = 0.024) (Table 1).

### Seasonal variation in gametocyte prevalence and density

Summary data for gametocyte prevalence and density are presented in Table 1, alongside the estimates from our statistical models, in which between-participant variation and parasite densities have been controlled for. The estimated gametocyte prevalence after drug treatment among the 25 participants, during transmission season 1 was 45.9% (s.e. = 4.35). Gametocyte prevalence varied across the seasons; this variation can be explained by seasonal variation in parasite density (parasite density: $X_{1}^{2}$ = 29.14, P < 0.001; season: $X_{3}^{2}$ = 8.592, P = 0.035; parasite density + season: $X_{3}^{2}$ = 3.33, P = 0.344).

**Fig 2:**
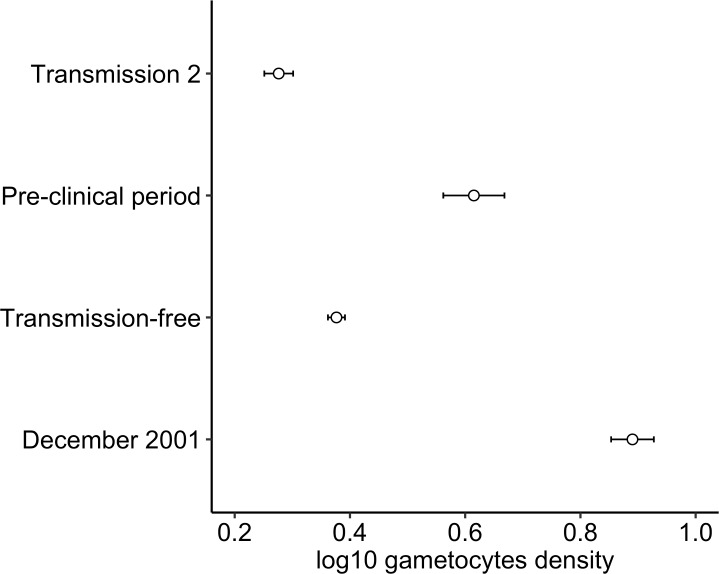
Association between gametocyte density and season. Gametocyte density, as predicted by the best-fit model, is significantly higher in the pre-clinical season compared to the transmission-free season and transmission season 2. Error bars represent the standard error of the mean. Gametocyte density is presented in arbitrary units, denoted as μL blood_**RNA**_ and is not directly comparable to total parasite density due to differences in sample processing and normalization to different WBC calibration curves.

**Fig 3:**
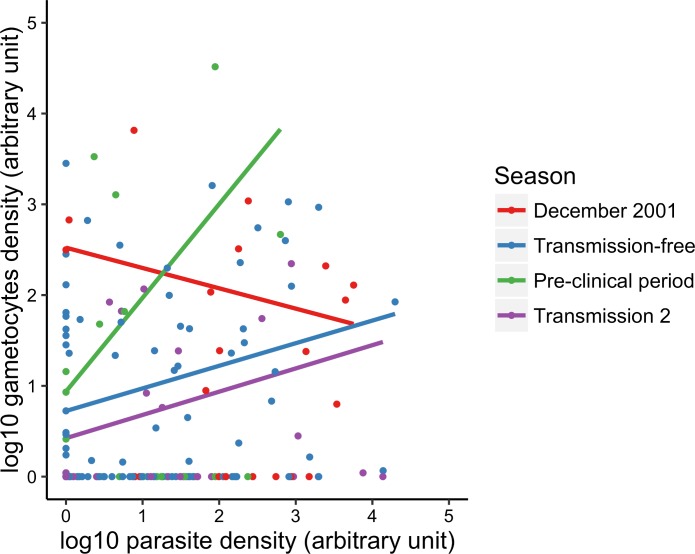
Correlation between parasite and gametocyte densities across seasons. The correlation between gametocyte and parasite densities varied across the seasons (i.e. there is a season by parasite density interaction). During the pre-clinical period, a strong and positive correlation was observed. This suggests that a larger proportion of total parasites are gametocytes during the pre-clinical season, compared to the other 3 seasons. Points represent raw data; lines represent the best-fit between values of log_10_ parasite and gametocytes densities as classified by season. Arbitrary numbers are used to present gametocyte and total parasite densities, denoted as blood_RNA_ and blood_DNA_, to account for sample processing differences that might result in the appearance that some samples contain more gametocytes than total parasites.

The average estimated gametocyte density after drug treatment during transmission season 1 (December 2001) was 0.89 log_10_ (se = 0.04) gametocytes/μl blood_RNA_. There was significant variation in gametocyte densities across the seasons ($X_{3}^{2}$ = 14.06, P = 0.003) (Fig 2); this variation was also associated with parasite densities ($X_{1}^{2}$ = 6.44, P = 0.011). There was a positive correlation between the densities of gametocytes and total parasites in the pre-clinical season compared to the transmission-free period (β estimate = 0.78, 95% CI = 0.13 to 1.43, P = 0.016; Fig 3). However, gametocyte densities were negatively correlated to total parasite densities in transmission season 1 (β estimate = -0.47, 95% CI = -0.93 to -0.02, P = 0.041; Fig 3), and there was no association with parasite density in transmission season 2 (β estimate = 0.01, 95% CI = -0.43 to 0.44, P = 0.974; Fig 3). After controlling for seasonal variation in parasite densities, estimated gametocyte densities decreased significantly from December 2001 to the transmission-free season ($X_{2}^{2}$ = 11.03, P = 0.004), then increased during the pre-clinical period ($X_{2}^{2}$ = 9.18, P = 0.010), and decreased during transmission season 2 ($X_{2}^{2}$ = 11.75, P = 0.003) (Fig 2).

## Discussion

The unique epidemiological setting of eastern Sudan, where malaria transmission occurs over a period of 8 to 10 weeks, and then ceases for 9 to 10 months, provides an opportunity to examine the hypothesis that seasonal variation in gametocyte density coincides with temporal fluctuations in transmission opportunities. This epidemiological setting spans the poor savannah belt of Sub-Saharan Africa [1,2,7–10], where annual rains are restricted to few months and the rest of the season remains hot and dry. Among the examined cohort, densities of *P*. *falciparum* total parasites and gametocytes fluctuated at sub-microscopic levels (0.64 log_10_ total parasites/μl blood_DNA_ and 0.36 log_10_ gametocytes/μl blood_RNA_, on average) in the transmission-free season between January and July 2002. However, following the start of the annual rains in the pre-clinical period (August to September 2002)—prior to the appearance of clinical cases—gametocyte densities increased while total parasite density remained at levels similar to the transmission-free season. This suggests an increase in gametocyte investment, which is further supported by the steeper correlation between gametocyte density and parasite density during the pre-clinical period compared to the transmission-free season.

The stability of parasite prevalence, density and gametocyte prevalence in the pre-clinical period compared to the transmission-free season suggests that the increase in gametocyte density is unlikely to be due to the acquisition of new infections. Instead, we suggest that *P*. *falciparum* parasites may respond to, or prepare for, the appearance of transmission opportunities by up-regulating investment into gametocytes (i.e. increasing conversion rate). Data from animal models shows that *P*. *relictum* responds to mosquitoes biting their avian host by increasing transmission potential [36] but whether these parasites detect mosquitoes directly (e.g. salivary proteins) or indirectly via a host reaction to bites (e.g. immune responses to salivary proteins) is unknown. *P*. *falciparum* may also respond to mosquito biting or, alternatively, respond to seasonal changes in host physiology. Seasonal changes occur in many aspects of human physiology and behaviour, including immune responses [45,46]. How parasites sense environmental change and translate this information into a change in phenotype is unclear but it has been suggested that Ca^2+^ dependent protein kinases are involved in activation of stage-specific development [47,48].

Variation in gametocyte density during infections has been linked to many in-host environmental and parasite genetic factors, including anti-malarial drug treatment [32,49,50], anaemia [25,27,28], and the presence of multiple genotypes within an infection [26]. Seasonal variation in these factors is likely to occur and could obscure or confound parasite responses to transmission opportunities. For example, new infections are more often symptomatic [2] and thus often result in drug treatment and/or anaemia [51]. New infections can also increase the multiplicity of existing infections [52] which has been linked to prolonged carriage of gametocytes [26]. We can probably rule out a direct impact of drug treatment as a confounding factor because participants who self-treated during June to September (prior and during the pre-clinical period) were excluded from analysis. All individuals received drug treatment at enrolment, and 20/25 participants’ received no further treatment during the transmission-free or the pre-clinical periods. However 5 individuals received self-treatment at the start of the transmission-free season, but none of them showed clinical symptoms beyond February 2002. The parasites in these individuals have apparently survived drug treatment, and could potentially be drug resistant. However, genotyping of drug resistance mutations (*pfcrt* codons 72–76 and *pfmdr1* codon 86) linked to chloroquine resistance showed a significant decrease in the proportion of drug-resistant parasite genotypes and parallel significant increase in wildtype from transmission season to the pre-clinical period among participants treated with chloroquine (data not shown). In the same period, gametocyte production increased and therefore, the pattern we observed cannot be attributed to increased gametocyte production by chloroquine resistant parasites. Anaemia and haemoglobin levels were not measured, and we cannot exclude potential effects of seasonal variation in haemoglobin levels that may occur due to e.g. diseases or physiological changes. However, if only malaria infections were to be considered, it is unlikely that haemoglobin levels would vary significantly between the transmission-free season and pre-clinical period as a result of sub-microscopic asymptomatic malaria infections. With regard to parasite multiplicity, it has been demonstrated that multiplicity among asymptomatic carriers can decrease over time [26]; we expect this to have remained stable during the relatively short period between the transmission-free season and pre-clinical period in the absence of new blood-stage infections. Therefore, we predict that seasonal variation in other aspects of the in-host environment or biting by mosquitoes provides parasites with more accurate information on transmission opportunities.

In addition to uncovering the information parasites may use, it is also necessary to test whether they respond in a manner that maximises fitness [53]. For example, does our observation of a decline in both total parasite and gametocyte densities from the pre-clinical period to transmission season 2 suggest that parasites prematurely increased gametocyte production? This is unlikely because a number of factors could reduce parasitaemia and gametocytaemia in the transmission season, including the acquisition of new infections that boost immune responses and the indiscriminate use of anti-malarials for any malaria-like symptoms [54,55]. Instead, we suggest that transmission of gametocytes occurs as soon as mosquitoes are available because the time frame required for gametocytes to be produced, mature, and undergo sporogony in mosquitoes, plus the exo-erythrocytic development of malaria in the host, corresponds to the lag between the appearance of mosquitoes and clinical cases [56].

Our main observations are supported by epidemiological surveys in other sites where annual rains and malaria transmission are short and highly seasonal [1,2,7–10], presenting circumstantial evidence for seasonality in the carriage of *P*. *falciparum* gametocytes [3,9,57,58]. For example, a longitudinal study in Burkina Faso found that season is an independent determinant of gametocyte prevalence and density and that it is significantly higher in the wet season compared to the dry season [9]. Furthermore, analysis of three historical data sets from different regions with seasonal malaria (Thailand, Tanzania and Nigeria) suggests that the intense seasonal pattern of uninfected mosquito bites during annual rains is associated with elevated gametocyte prevalence and can ignite transmission [59]. More recently, it has been shown that gametocytes produced at the start of the transmission season in Burkina Faso are more infectious to mosquitoes than those during the peak of the transmission season or the dry season [56].

In our study we have observed similar gametocyte prevalence during the transmission-free and the pre-clinical periods. Indeed, gametocytes must be present to obtain any transmission, however increasing gametocyte densities generally improves transmission success particularly at very low gametocytaemia [60,61] and therefore also represents a fitness benefit to the parasite. The increase in gametocyte density but not prevalence can be attributed to three main factors, as the following: (a) the small cohort and the possibility that not all infections (isolates) are committed to gametocyte production (3 participants did not show gametocytes throughout the study period). Early *in vitro* studies suggested that *P*. *falciparum* isolates could vary significantly in their capacity to produce gametocytes [18,62]. (b) Due to periodicity of gametocytogenesis we may not have picked up all gametocyte producer infections at all sampling times. (c) Owing to the highly sensitive methods used for detection, the proportion of samples with gametocytes below the detection limit is small in all seasons, and therefore gametocyte prevalence is unlikely to be statistically different between seasons.

Some limitations of our study include first, our sampling method precluded estimates of the gametocyte conversion rate. Estimating conversion rates remains inherently difficult in natural *Plasmodium* infections, because of overlapping cohorts of gametocytes circulating at the same time, which makes it difficult to establish which proportion of gametocytes originates from a specific asexual replication cycle 10–14 days earlier [63]. Instead we have taken a commonly used approach and investigated the association between gametocyte and total parasite dynamics. The increase in gametocyte densities in absence of changes in asexual densities from the transmission-free to the pre-clinical season, and the observed steeper slope of the correlation between total parasite and gametocyte densities support the hypothesis of increased gametocyte production during the pre-clinical period. Second, entomological data were not collected during this study and the seasons have been defined based only on weather data (rains and temperature). However, the weather data were consistent with previous reports in the study village, in which a uniform pattern of annual rains and appearance of mosquitoes was shown [2]. We therefore believe that our definition of season, based on weather data, is sufficient to link the pre-clinical period (August—September) to the expected appearance of mosquitoes. Third, sample sizes were limited by ethical constraints and the low prevalence of infection in the area. Limited sample sizes, combined with inter-assay variability in qPCR quantification of parasites, can make data analysis difficult. However, these issues are reflected in our error estimates, and did not prevent the detection of a significant pattern.

In summary, in eastern Sudan, an area of distinct seasonal transmission, *P*. *falciparum* sustains chronic asymptomatic infections throughout the transmission-free season, characterised by low parasite densities, but producing gametocytes. Despite, some limitations to our study, we have shown that gametocyte production increases after the transmission-free season. This suggests that in areas of seasonal malaria, the parasite may have evolved to recognise vector abundance, or a proxy for it, and responds in a manner that maximises transmission opportunities. Control measures aiming at reducing asymptomatic carriage, during the transmission-free season, should therefore have a significant impact on control of cyclical malaria in such areas.
